# Restoration of ceramide *de novo* synthesis by the synthetic retinoid ST1926 as it induces adult T-cell leukemia cell death

**DOI:** 10.1042/BSR20200050

**Published:** 2020-10-28

**Authors:** Botheina Ghandour, Claudio Pisano, Nadine Darwiche, Ghassan Dbaibo

**Affiliations:** 1 Department of Biochemistry and Molecular Genetics, American University of Beirut, Lebanon; 2 Biogem Research Institute, Italy; 3 Department of Pediatrics and Adolescent Medicine, Center for Infectious Diseases Research, American University of Beirut, Lebanon

**Keywords:** Adamantyl retinoid, Adult T cell leukemia, Ceramide, ST1926, T lymphoma

## Abstract

Ceramide (Cer) is a bioactive cellular lipid with compartmentalized and tightly regulated levels. Distinct metabolic pathways lead to the generation of Cer species with distinguishable roles in oncogenesis. Deregulation of Cer pathways has emerged as an important mechanism for acquired chemotherapeutic resistance. Adult T-cell leukemia (ATL) cells are defective in Cer synthesis. ATL is an aggressive neoplasm that develops following infection with human T-cell lymphotropic virus-1 (HTLV-1) where the viral oncogene Tax contributes to the pathogenesis of the disease. ATL cells, resistant to all-*trans*-retinoic acid, are sensitive to pharmacologically achievable concentrations of the synthetic retinoid ST1926. We studied the effects of ST1926 on Cer pathways in ATL cells. ST1926 treatment resulted in early Tax oncoprotein degradation in HTLV-1-treated cells. ST1926 induced cell death and a dose- and time-dependent accumulation of Cer in malignant T cells. The kinetics and degree of Cer production showed an early response upon ST1926 treatment. ST1926 enhanced *de novo* Cer synthesis via activation of ceramide synthase CerS(s) without inhibiting dihydroceramide desaturase, thereby accumulating Cer rather than the less bioactive dihydroceramide. Using labeling experiments with the unnatural 17-carbon sphinganine and measuring the generated Cer species, we showed that ST1926 preferentially induces the activities of a distinct set of CerS(s). We detected a delay in cell death response and interruption of Cer generation in response to ST1926 in Molt-4 cells overexpressing Bcl-2. These results highlight the potential role of ST1926 in inducing Cer levels, thus lowering the threshold for cell death in ATL cells.

## Introduction

Retinoids are powerful compounds known for their tumor-suppressive roles as they regulate hematopoietic cell proliferation and differentiation [1–4]. Although all-*trans* retinoic acid (ATRA), the active metabolite of vitamin A, is used for the treatment of certain leukemia types particularly acute promyelocytic leukemia (APL), the use of natural retinoids is hindered by acquired resistance and side effects [5,6]. The retinoid related molecules (RRMs), such as N-(4-hydroxyphenyl) retinamide (HPR), 6-[3-(1-adamantyl)-4-hydroxyphenyl]-2-naphthalene carboxylic acid (CD437), and (2E)-3-[3′-(1-adamantyl)-4′-hydroxy[1,1′-biphenyl]-4-yl]-2-propenoic acid (ST1926) show potent anti-neoplastic activities [5]. In adult T-cell leukemia (ATL), both HPR and CD437 induce growth arrest and cell death in human T-cell lymphotropic virus-1 (HTLV-1) positive and negative T cells, including those that are ATRA-resistant [7,8]. However, treatment with ST1926 requires lower doses and shows potent antitumor effects with minimal toxicity, increased specificity, and broad spectrum of activity in solid and hematological malignancies *in vivo* and *in vitro*, including many that are ATRA-resistant [9,10]. In addition to inducing DNA damage and S-phase arrest, working through RAR-dependent and independent mechanisms [11], both CD437 and ST1926 target DNA polymerase 1 alpha (POLA1) [3,12]. We have previously reported that ST1926 is a more potent inducer of growth inhibition and massive apoptosis than HPR or CD437, especially in HTLV-1 positive T cells, probably due to the fact that only ST1926 causes early down-regulation of Tax, the critical oncoprotein of HTLV-1 [1,8]. ST1926 also prolongs survival and reduces leukemic cell infiltration in ATL mice, decreases Tax mRNA and DNA, and induces apoptosis *in vivo* [1]. Meanwhile, Tax oncoprotein protected cells from ceramide (Cer) accumulation and apoptosis [8,13]. Given the fact that ATL, similar to most blood cancers, displays a genetically heterogeneous clonal profile and due to chemotherapy resistance, alternative therapies that could be mediated by the accumulation of lethal levels of Cer might have potential promise [14–17].

Cer, a sphingolipid-tumor suppressor, acts as a second messenger that mediates cell death and growth suppression through several mechanisms such as apoptosis, necroptosis, senescence, cell cycle arrest, and autophagy [18–21]. Cer pathways are highly conserved and act as coordinators of eukaryotic stress response [22]. A variety of signals could trigger Cer production, including chemotherapeutic agents [23,24]. Numerous studies have identified direct Cer targets, namely Cer-activated protein phosphatases (CAPPs), which constitute serine/threonine protein phosphatases PP1 and PP2A [25]. These phosphatases act on several substrates that promote changes in growth arrest, apoptosis, and/or senescence, such as retinoblastoma gene product RB, Bcl-2, AKT, and c-Jun [22,26,27]. Cer production is highly compartmentalized and occurs via three main metabolic pathways, through *de novo* synthesis, sphingomyelin turnover, or the salvage pathway following the reacylation of sphingosine generated from hydrolyzed Cer that is in turn generated from the hydrolysis of more complex sphingolipids [19,28,29]. Alternatively, Cer could be also generated by the inhibition of its metabolism by glucosyl-ceramide synthase (GCS) and/or sphingomyelin synthase (SMS), or its clearance by ceramidase (CDase) [30–32]. Indeed, acid CDase was found to be overexpressed in acute myeloid leukemia (AML) patients [16], modulating Mcl-1 expression, while its inhibition sensitizes cells to chemotherapeutics [31,33]. Moreover, using acid CDase inhibitor along with HPR treatment sensitizes human prostate cancer cells to apoptosis [34]. GCS was found to be overexpressed in several types of leukemic cell lines including those that are chemotherapy resistant [30,35,36], and maintains a potential role in lymphoma and myeloma tumor initiation [37]. Indeed, inhibiting GCS by 1-phenyl-2-decanoylamino-3-morpholino-1-propanol (PDMP) and the subsequent Cer accumulation sensitize imatinib-resistant chronic myeloid leukemia cells among other types of cancer cells [36,38]. Moreover, blocking Cer glucosylation with PDMP synergizes with HPR treatment to induce cell death in HTLV-1 positive human T-cells [13]. We have previously shown that HPR increases Cer levels in HTLV-1 negative leukemia cells, but not in HTLV-1 transformed cells, concomitant with lower sensitivity of HTLV-1 positive cells to treatment with exogenous C2- and C6-Cer. This was attributed to a defect in *de novo* Cer synthesis in HTLV-1 positive cells treated with HPR. Indeed, Tax protein transfected cells were less sensitive to HPR-induced cytotoxicity and generated lower levels of Cer. In fact, targeting Cer metabolism might alter drug resistance in a variety of solid tumors and hematological malignancies [39].

Also known as N-acylsphingosine, Cer consists of a C18-sphingoid base backbone to which fatty acid chains of variable lengths are added [19,40], whereby the most commonly found Cer species in mammalian cell membranes are with C16-C24 fatty acyl chains [34]. Based on the lengths of different fatty acyl chains they utilize in the *de novo* pathway, six different ceramide synthases (CerSs) have been discovered that are products of different genes [17,19,41,42]. The specificity of these enzymes includes substrate preference for different fatty acyl chain lengths generating the corresponding Cer species, which vary in their tissue and subcellular localization, context of stimulation, and availability of downstream targets that ultimately dictates distinct roles in cancer cell death and/or survival [19,43,44]. The chain length specific effects of Cer have gained more attention in tumorigenesis, as high-throughput, structural, and quantitative analytical methods allowed for better understanding of the corresponding CerS(s) spatiotemporal regulation and role in cancer cell killing [45]. As for the spatial regulation, it has been recently shown that CerS(s) can localize in the mitochondrial outer membrane, which leads to Cer channel formation prior to the induction phase of apoptosis and facilitates the release of pro-apoptotic proteins from the intermembrane space into cytoplasm [46,47]. Cancer lipidomics is emerging as a new cancer profiling method to monitor prognosis, diagnosis, and treatment [48]. There is solid evidence for dysregulation of sphingolipid metabolism in hematological malignancies which often confer resistance to current treatment regimens [17]. Thus, the promise of combining sphingolipid modulators with chemotherapeutics would provide novel approaches for treating several blood cancers.

In the present work, we demonstrate that clinically achievable concentrations of ST1926 provoke similar Cer responses, in both HTLV-1 positive and negative malignant T cells, which was not observed with previously tested RRMs such as HPR and CD437. ST1926 but not HPR or CD437 causes early Tax degradation. Compared with HPR, which accumulates dihydroceramide (dhCer), ST1926 enhances *de novo* Cer synthesis via activation of specific CerS(s) without inhibiting dihydroceramide desaturase (DEGS1), thereby accumulating Cer rather than dhCer. At the mitochondrial level, the intrinsic pathway of apoptosis is tightly regulated by the Bcl-2 family members and orchestrated by the bioactive sphingolipid ceramide. At this level, the pro-apoptotic Cer might antagonize the effects of Bcl-2 oncoprotein in a multitude of pathways. We dissected the interplay between the anti-apoptotic protein Bcl-2 and Cer. We observed that Bcl-2 attenuated ST1926 growth-inhibitory response and interrupted both *de novo* Cer accumulation and cell death in Molt-4 cells. These results highlight the potential role of ST1926 in inducing Cer levels, thus lowering the threshold for cell death in ATL cells and overcoming resistance.

## Materials and methods

### Cell lines, drugs, and culture conditions

The HTLV-1 transformed CD4+ T cell-lines, HuT-102 and MT-2, and HTLV-1 negative CD4+ T- cell lines, Jurkat and Molt-4 were grown as previously described [7]. Molt-4 cells transfected with p-MEP4 with (Molt4-Bcl2) or without (Molt4-MEP4) full-length murine *bcl-2* were clonally selected for once per week and before each experiment with Hygromycin B1 (Invitrogen) (500 μg/ml). ST1926 was prepared as stock solutions in dimethylsulfoxide (DMSO) at 1 × 10^−2^ M in amber tubes and stored at −80°C. All cells were grown in RPMI-1640 (Lonza) medium containing 10% (v/v) Fetal Bovine Serum (FBS) and antibiotics. All experiments were done under dim light. A seeding density of 3 × 10^5^ cells/ml was chosen for all experiments, unless specified otherwise. The synthetic retinoid, ST1926 was kindly provided by Sigma-Tau and Biogem (Ariano Irpino, Italy) and reconstituted in DMSO at a concentration of 10^−2^ M and stored at −80°C. The final concentrations of DMSO never exceeded 0.1%, which showed no effect on the proliferation of all tested cells. The unnatural 17C-sphinganine was purchased from Avanti Polar Lipids and reconstituted in DMSO to be added at a concentration of 4 μM.

### Ceramide measurement

Lipids were collected by the method of Bligh and Dyer [49]. Cer was measured with a modified diacylglycerol kinase (DGK) assay using external standards as described previously [50]. Briefly, 80% of the lipid sample was dried under N_2_. The dried lipid was solubilized in 20 μl of an octyl β-D-glucoside/dioleoyl phosphatidylglycerol micellar solution (7.5% octyl β-D-glucoside, 25 mM dioleoyl phosphatidylglycerol) by several cycles of sonication for 30 min. The reaction buffer was prepared as a 2× solution, containing 100 mM imidazole HCl (pH 6.6), 100 mM LiCl, 25 mM MgCl_2_, and 2 mM EGTA. To the lipid micelles, 50 μl of 2× reaction buffer were added, 0.2 μl of 1 M dithiothreitol, 5 μg of diglycerol kinase membranes and dilution buffer (10 mM imidazole, pH 6.6, 1 mM diethylenetriaminepenta-acetic acid, pH 7) to a final volume of 90 μl. The reaction was started by adding 10 μl of 2.5 mM [γ -**^32^**P] ATP solution (specific activity of 75,000–200,000 cpm/nmol). The reaction was allowed to proceed at 25°C for 30 min. Bligh and Dyer lipid extraction was performed and a 1.5 ml aliquot of the organic phase was dried under N_2_. Lipids were then resuspended in 50 μl of methanol/chloroform (1:9, v/v) and 25 μl was spotted on to a 20 cm silica gel TLC plate. Plates were developed using chloroform/acetone/methanol/acetic acid/H_2_O (50:20:15:10:5, by vol.), air-dried and subjected to autoradiography. The radioactive spots corresponding to phosphatidic acid and ceramide-phosphate, the phosphorylated products of diacylglycerol and Cer, respectively, were identified by comparison with known standards. Spots were scraped into a scintillation vial containing 4 ml of scintillation fluid and counted in a scintillation counter. Linear curves of phosphorylation were produced over a concentration range of 0–960 pM of external standards (CIII-ceramide, Sigma). Cer levels were routinely normalized to lipid phosphate levels. It is important to note that under these conditions, there was a total conversion of Cer and diacylglycerol into their phosphorylated products, and there was no change in the specific activity of the DGK enzyme. Cer levels were normalized to lipid phosphate levels.

### *De novo* ceramide synthesis

At initiation of treatment, [^3^H]-palmitic acid (1 μCi/ml medium) purchased from Perkin Elmer (32.0 Ci/mmol) was added to treated and untreated samples. At the indicated time points, lipids were extracted according to Bligh and Dyer method [49], N_2_ dried, and resuspended in 60 μl chloroform:methanol (2:1); 40 μl were spotted on 20 cm silica gel TLC plates. Plates were developed with ethylacetate:isooctane:acetic acid (90:50:20, v/v), air dried, and sprayed lightly with En3hance® (Perkin Elmer) to enhance tritium readings. [^3^H]-Cer spots were visualized by iodine vapor mark. Radioactivity was visualized by autoradiography after 96 h at −80°C and the [^3^H]-Cer spots were scraped into scintillation vials containing 4 ml of scintillation fluid and counted on a Packard scintillation counter. [^3^H]-Cer counts were normalized to lipid phosphate levels.

### *In vitro* ceramide synthase and dihydroceramide desaturase activity

Cells were treated with unnatural 17C-sphinganine and either 0.1% DMSO or 1 μM ST1926 for 24 h. Activities of the mentioned enzymes were determined using lyophilized samples from ST1926 treated and untreated cells. The subsequently generated unnatural 17C-dihydroceramide (17dhCer) and 17C-ceramide (17Cer) species levels were identified and quantified using LC-MS and then normalized to total lipid phosphates to reflect the activities of the corresponding CerS(s) and DEGS1, respectively.

### Liquid chromatography-mass spectrometry

Cells were harvested at the indicated time-points, and cell pellets were washed twice with 1× PBS, and shipped as lyophilized samples to the Medical University of South Carolina (MUSC) for sphingolipidomics analysis (LC/MS) as described [51]. Samples were fortified with internal standards and lipids were extracted with ethyl acetate/isopropanol/water (60:30:10 by vol.), evaporated to dryness and reconstituted in 100 μl of methanol. Analysis was performed using electrospray ionization MS/MS analysis on a Thermo Finnigan TSQ 7000 triple quadruple mass spectrometer, operating in multiple-reactions-monitoring positive-ionization mode, as previously described [52].

### Immunoblot analysis

Total protein extracts from treated and untreated cells were prepared as previously described [8]. GAPDH (MAB5476) (Abnova, Heidelberg, Germany) was used as control. Mouse monoclonal anti-Tax (168-A51) was obtained from the National Institutes of Health AIDS Research and Reagent Program. Rabbit polyclonal anti-CerS2 antibody (ab227501) and rabbit polyclonal anti-LASS6/CerS6 (ab115539) were obtained from abcam.

### RNA extraction

Cells were lysed and total RNA isolated using TRI Reagent (Sigma Aldrich) according to the manufacturer’s protocol and quantified using DeNovix DS-11FX Spectrophotometer according to the manufacturer’s protocol.

### cDNA synthesis and quantitative real-time polymerase chain reaction (qRT-PCR)

RNA samples were reverse-transcribed using QuantiTect Reverse Transcription Kit (QIAGEN, catalogue #205311) according to the manufacturer’s instructions. Primer sequences for the three CerS genes and for the housekeeping gene β-actin are: **CerS2**, 5′-CCG ATT ACC TGC TGG AGT CAG-3′ (Forward), and 5′-GGC GAA GAC GAT GAA GAT GTT G -3′ (Reverse); **CerS4**, 5′-CTT CGT GGC GGT CAT CCT G-3′ (Forward), and 5′-TGT AAC AGC AGC ACC AGA GAG-3′ (Reverse); **CerS6**, 5′-GGG ATC TTA GCC TGG TTC TGG-3′ (Forward), and 5′-GCC TCC TCC GTG TTC TTC AG-3′ (Reverse); β**-actin**, 5′-ATT GGC AAT GAG CGG TTC C-3′ (Forward), and 5′-GGT AGT TTC GTG GAT GCC ACA-3′ (Reverse). Briefly, cDNA was amplified in a 20 μl mixture loaded in 96-well plates containing forward and reverse primers, SYBR® Green JumpStart Taq ReadyMix (Sigma-Aldrich, catalogue #S4438) and RNase-free water. The thermal cycling conditions consisted of an initial denaturation step at 95°C for 15 min, followed by 40 cycles of denaturation at 95C for 15 s, annealing for 45 s and extension at 72°C for 1 min. A melt curve was incorporated at the end of each reaction to ensure the specificity of the product. Relative expression analysis was performed using the 2^−ΔΔCt^ calculation method by normalization to the housekeeping gene β-actin.

## Results

### ST1926 induces early ceramide accumulation in both HTLV- 1 positive and negative malignant T cells

**Figure 1:**
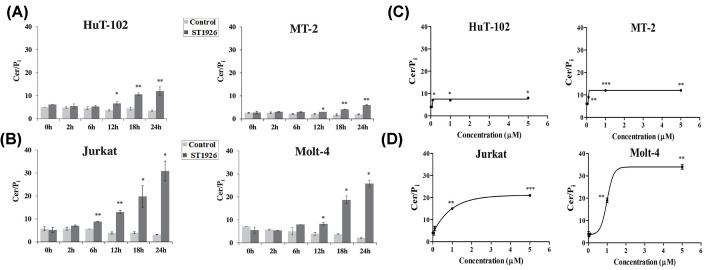
ST1926 treatment causes early time- and dose-dependent accumulation of ceramide in HTLV-1 positive and negative malignant human T cells (**A**) Ceramide (Cer) levels in HTLV-1 positive (HuT-102 and MT-2) and (**B**) HTLV-1 negative (Jurkat and Molt-4) human T-cell lines. Cells were seeded at a density of 3 × 10^5^ cells/ml and treated with 0.1% DMSO as control or with 1 μM ST1926 for the times indicated. Cer levels were determined in triplicates using the DGK assay as described in the ‘Materials and Methods’ section and normalized to total cellular lipid phosphate levels. Data points represent the mean (± SD). Results are representative of two independent experiments. (**C**) Dose–response to ST1926 treatment in HTLV-1 positive (HuT-102 and MT-2) and (**D**) HTLV-1 negative (Jurkat and Molt-4). Cells were seeded at a density of 3.5×10^5^ cells/ml and treated with 0.1% DMSO as a control, or the indicated concentrations of ST1926, for 24 h. Cer levels were determined as in (A and B). Data points represent the mean (± SD). Results are representative of two independent experiments. The asterisks *, **, and *** indicate statistically significant differences at *P*≤0.05, *P*≤0.01, or P≤0.001 respectively, versus control using the *t*-test.

We have previously determined that HPR produces distinct Cer responses in HTLV-1 positive and HTLV-1 negative malignant T cells, whereby Tax-transformed T cells showed a defect in accumulating Cer [13]. Therefore, we tested the effect of ST1926 on the kinetics of Cer accumulation in HTLV-1 positive (HuT-102 and MT-2) and HTLV-1 negative (Molt-4 and Jurkat) malignant T cell lines. We selected an ST1926 concentration of 1 μM, as it results in more than 90% of growth inhibition at 48 h in all tested cells, with no effect on resting or activated peripheral blood mononuclear cells [1]. Treatment with this dose generated a time-dependent response in Cer in HTLV-1 positive cells (Figure 1A). Cer accumulation started at 12 h, reaching 3-fold of baseline by 24 h in HuT-102 and MT-2 cells. In HTLV-1 negative cells, treatment with 1 μM ST1926 resulted in a similar, but earlier and more pronounced accumulation of Cer (Figure 1B). The significant rise in Cer levels became apparent by 6 h in Jurkat and by 12 h in Molt-4 cells with 2-fold increase, reaching at least 10-fold by 24 h in both cell types (Figure 1B). Notably, the sustained increase in Cer levels preceded major ST1926-induced growth suppression and cell death in all tested HTLV-1 positive and negative cells at the indicated concentrations [1]. Furthermore, we observed a dose-dependent response of Cer accumulation in all tested cells using ST1926 concentrations ranging from 0.05 μM up to 5 μM for 24 h. Cer increase reached 2-fold of baseline levels with concentrations of ST1926 as low as 0.1 μM in HTLV-1 positive cells (Figure 1C). Meanwhile, Cer accumulation reached 5- and 3-fold of baseline in Molt-4 and Jurkat cells respectively when the concentration was increased to 1 μM (Figure 1D). Our results show a dose-dependent accumulation of Cer in both ST1926-treated HTLV-1 positive and negative T cells.

### ST1926 induces early *de novo* ceramide synthesis in HTLV-1 positive and negative malignant T cells

**Figure 2:**
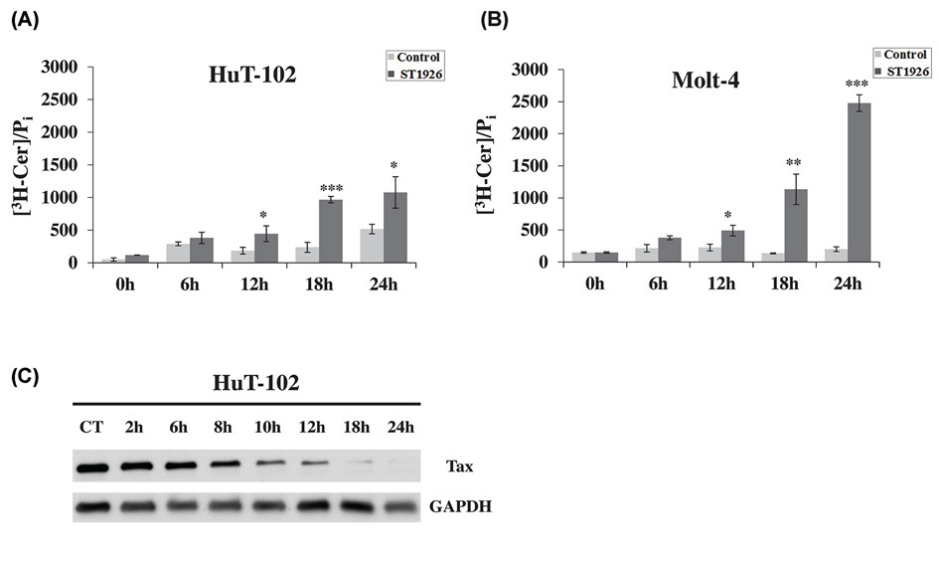
ST1926 stimulates early *de novo* ceramide synthesis in HTLV-1 positive and negative malignant human T cells (**A**) ST1926 induces *de novo* ceramide (Cer) production in HTLV-1 positive (HuT-102) and (**B**) HTLV-1 negative (Molt-4) malignant T-cells. HuT-102 and Molt-4 cells were seeded at a density of 3 × 10^5^ cells/ml and treated with 0.1% DMSO as control or 1 μM ST1926 for the times indicated. *De novo* Cer levels were determined in triplicates using the [^3^H]-palmitate incorporation method as described in ‘Materials and Methods’ section and normalized to total cellular lipid phosphate levels. Data points represent the mean (± SD). Results are representative of two independent experiments. The asterisks *, **, and *** indicate statistically significant differences at *P*≤0.05, *P*≤0.01, or *P*≤0.001, respectively, versus control using the *t*-test. (**C**) ST1926 causes early degradation of Tax oncoprotein levels in HuT-102 cells. Cells were seeded at a density of 2 × 10^5^ cells/ml and treated with 0.1% DMSO as control or 1 μM ST1926 for the times indicated. Whole SDS protein lysates (50 μg/lane) were prepared and immunoblotted against Tax antibody. The blot was re-probed against GAPDH to ensure equal protein loading.

We have previously shown that HTLV-1 positive cells have impaired Cer production in response to HPR, such that *de novo* Cer synthesis is only induced in treated HTLV-1 negative cells [13]. Moreover, the defect in Cer synthesis upon HPR treatment is Tax-dependent in HTLV-1 positive cells, as well as in Tax over-expressing Molt-4 and HeLa cells. Therefore, we examined the contribution of *de novo* Cer synthesis in HuT-102 and Molt-4 cells in response to ST1926 in order to elucidate the role of this pathway in the resulting Cer accumulation in HTLV-1 positive and HTLV-1 negative malignant T cells, respectively. HuT-102 and Molt-4 cells were treated with 1 μM ST1926 at the indicated time-points, and *de novo* synthesized Cer was measured by quantifying [**^3^**H]-palmitate incorporation into newly synthesized Cer following treatment. ST1926 treatment caused an early time-dependent increase in [^3^H]-Cer in both cell lines, as early as 12 h post treatment (Figure 2A,B). ST1926-induced *de novo* synthesis was more pronounced in Molt-4 cells reaching about 10-fold increase over baseline levels versus a 2-fold increase in HuT-102 cells at 24 h post treatment (Figure 2A,B). Interestingly, *de novo* Cer accumulation in HuT-102 started at 12 h and was preceded by Tax protein degradation in HuT102 that was demonstrable by 8 h (Figure 2C).

### Ceramide, but not dihydroceramide, species predominantly accumulate in response to ST1926 treatment in HTLV-1 positive and negative malignant T cells

We have previously shown that HTLV-1 positive and HTLV-1 negative cells showed distinct Cer responses upon treatment with HPR, whereby Tax overexpression alone in cells not infected with HTLV-1 was sufficient to suppress *de novo* Cer synthesis [13]. It was later reported that DEGS1, the enzyme responsible for converting dhCer to Cer, is directly inhibited by HPR leading to the accumulation of endogenous dhCers rather than Cers [53]. Although dhCer, unlike Cer, has been considered biologically inactive in the context of cell death induction [54], there is evidence that it might rather have an inhibitory role [55]. Stiban et al. showed that dhCer interferes with Cer channel formation in the mitochondria, consequently inhibiting permeabilization and transition to the apoptotic response. In fact, reports demonstrate opposing roles of DEGS1 in apoptosis [56], whereby its polyubiquitination results in ‘gain of function’ with pro-survival effects [57]. Indeed, inhibiting DEGS1 activity with drugs other than HPR, siRNA, or gene depletion causes resistance to apoptosis by various stimuli [58–61]. Meanwhile, depending on cell types, dhCer might not have a role in HPR-triggered apoptosis [62], suggesting that the apoptogenic potential depends on the ratio of dhCer to Cer, rather than dhCer alone [55,62].

**Figure 3:**
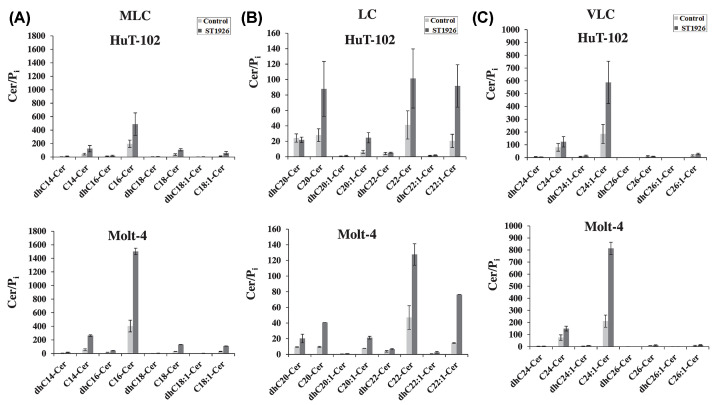
Accumulation of ceramide, but not dihydroceramide species in HTLV-1 positive and negative malignant T cells in response to treatment with ST1926 (**A**) Medium long-chain (MLC), (**B**) long-chain (LC), and (**C**) very long-chain (VLC) dihydroceramide (dhCer) or ceramide (Cer) species levels in HTLV-1 positive (HuT-102) and HTLV-1 negative (Molt-4) cells. Cells were seeded at a density of 3 × 10^5^ cells/ml and treated with 0.1% DMSO as control or 1 μM ST1926 for 24 h. DhCer/Cer species levels (pmol) were measured in lyophilized samples in duplicates by LC/MS as described in ‘Materials and Methods’ section and normalized to total cellular lipid phosphate levels (μmol). Data points represent the mean ± range (*n*=2). Results are representative of two independent experiments.

In order to determine whether the effects of ST1926 on the regulation of Cer metabolism were similar to, or distinguishable from, those of HPR, we measured various Cer and/or dhCer species that are generated upon ST1926 treatment in HTLV-1 positive and negative malignant T-cells as represented by HuT-102 and Molt-4 cells, respectively. Cer and/or dhCer species were identified based on the fatty acyl-chain length that is preferentially introduced by different CerS(s) on the sphingoid base backbone of sphinganine. In this regard, the resulting Cer species were categorized as medium long-chain (MLC: C14- C18), long-chain (LC: C20- C22), and very long-chain (VLC: C24-C26). In HuT-102 cells, Cer+dhCer levels increased from 738 to 1923 pmol/μmol, which represents an increase by 1185 pmol/μmol upon treatment with 1 μM ST1926 for 24 h (Table 1A). In Molt-4 treated cells, Cer+dhCer levels increased from 947 to 3369 pmol/μmol, which represents an increase by 2422 pmol/μmol. Overall, MLC species accounted for most of the accumulation of the total dhCer and Cer levels in both cell lines, followed by VLC species, with the least contribution by the LC species (Table 1B). ST1926 treatment resulted in a major accumulation of Cer species relative to dhCer species in both HuT102 and Molt-4 cells Importantly, Cer elevation was found to be consistent among most MLC (Figure 3A), LC (Figure 3B), and VLC (Figure 3C) Cer species. For instance, among MLC Cers, accumulation of C16-Cer represented the most prominent increase among other species in this category in both HuT-102 and Molt-4 treated cells (Figure 3A). Similarly, there was a total increase in LC Cers, majorly represented by C22 and C22:1-Cer species, among others in both cell lines (Figure 3B). Consistently, VLC Cers had similar trends of increasing levels in both HuT-102 and Molt-4 cells, with C24:1-Cer being the most dominant in this category (Figure 3C). This analysis supports the conclusion that ST1926 induces early *de novo* Cer synthesis in both cell lines without appreciable accumulation of dhCer suggesting a lack of inhibition of DEGS1 by ST1926.

**Table 1:** Percent accumulation of total and specific fatty-acyl chain ceramide (Cer) and dihydroceramide (dhCer) species in HTLV-1 positive and negative malignant T cells upon treatment with ST1926

	Total (dhCer + Cer)	Ceramide (% Total Cer)	Dihydroceramide (% Total Cer)	
**(A)** Percent increase in total Cer and dhCer species in HuT-102 and Molt-4 cells				
HuT-102	1185	98	2	
Molt-4	2422	97	3	

	Total (dhCer + Cer)	MLC Cer (% Total Cer)	LC Cer (% Total Cer)	VLC Cer (% Total Cer)
**(B)** Percent increase of MLC, LC, and VLC Cer and dhCer species in HuT-102 and Molt-4 cells				
HuT-102	1185	43	18	39
Molt-4	2422	63	8	29

**(A)** Percent increase in total Cer and dhCer species in HTLV-1 positive (HuT-102) and negative (Molt-4) cells. (**B**) Percent increase of medium long chain (MLC), long chain (LC), and very long chain (VLC) Cer and dhCer species in HuT-102 and Molt-4 cells. Cells were seeded at a density of 3 × 10^5^ cells/ml and treated with 0.1% DMSO as control or 1 μM ST1926 for 24 h. DhCer/Cer species levels (pmol) were measured in lyophilized samples as duplicates by LC/MS as described in ‘Materials and Methods’ section and normalized to total cellular lipid phosphate levels (μmol). Data points represent the mean ± range (*n*=2). Results are representative of two independent experiments.

### Treatment with ST1926 activates ceramide synthases in both HTLV-1 positive and negative leukemic T cells

**Figure 4:**
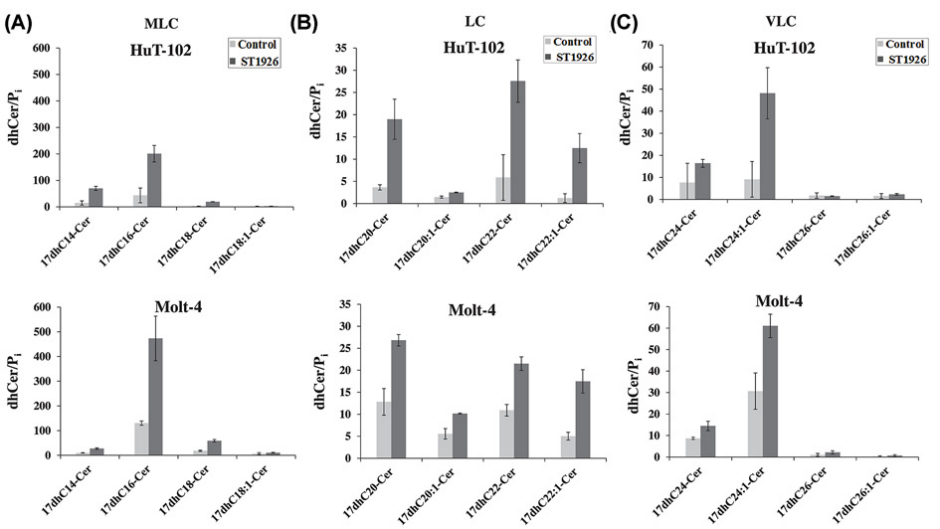
ST1926 activates ceramide synthase in HTLV-1 positive and negative malignant T cells (**A**) Medium long-chain (MLC), (**B**) Long-chain (LC), and (**C**) Very long-chain (LC) 17C-dihydroceramide (17dhCer) species levels in HTLV-1 positive (HuT-102) and HTLV-1 negative (Molt-4) cells. Cells were seeded at a density of 3 × 10^5^ cells/ml, labeled with 4 μM of unnatural 17C-sphinganine and treated with either 0.1% DMSO as control or 1 μM ST1926 for 24 h. 17dhCer species levels (pmol) were measured in lyophilized samples in duplicates by LC/MS as described in ‘Materials and Methods’ section and normalized to total cellular lipid phosphate levels (μmol). Data points represent the mean ± range (*n*=2). Results are representative of two independent experiments.

**Figure 5:**
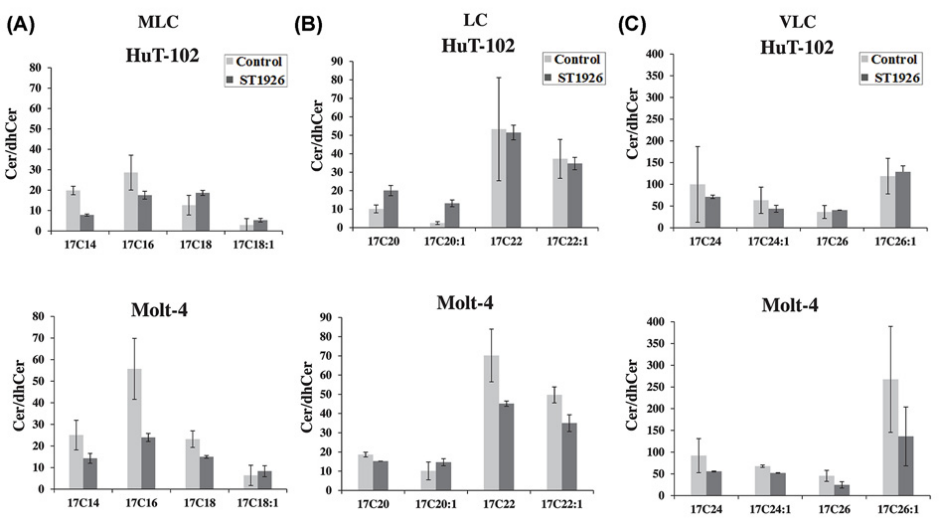
Effect of ST1926 on the activity of dihydroceramide desaturase (DEGS1) in HTLV-1 positive and negative malignant T cells (**A**) Medium long-chain (MLC), (**B**) long-chain (LC), and (**C**) very long-chain (VLC) 17C-ceramide (17Cer) species levels in HTLV-1 positive (HuT-102) and HTLV-1 negative (Molt-4) cells. Cells were seeded at a density of 3 × 10^5^ cells/ml, labeled with 4 μM of unnatural 17C-sphinganine and treated with either 0.1% DMSO as control or 1μM ST1926 for 24 h. 17Cer species levels (pmol) were measured in lyophilized samples as duplicates by LC/MS as described in ‘Materials and Methods’ section, and the activity of DEGS1 was determined by the ratio of 17Cer to 17C-dihydroceramide (17dhCer) levels. Data points represent the mean ± range (*n*=2). Results are representative of two independent experiments.

Previously, we have shown that Tax expression in cells not infected with HTLV-1 was sufficient to inhibit the generation of Cer in response to HPR and to suppress *de novo* Cer synthesis, probably due to Tax inhibitory effect on CerS activity [13]. To further characterize Tax-dependent regulation of *de novo* Cer synthesis and to identify the enzymes involved in HTLV-1 positive and negative leukemic T cells in response to ST1926, we treated cells labeled with the unnatural 17C-sphinganine to indirectly determine the activities of CerS(s) and DEGS1. Measurement of the resulting unnatural endogenous 17dhCer and 17Cer species, the products of the acylation followed by desaturation reactions of the 17C-sphinganine by CerS(s) and desaturase, respectively, were obtained using LC-MS analysis following treatment with ST1926 for 24 h in HuT-102 and Molt-4 cells. In HuT-102 cells, we observed a 5-fold increase above baseline in each of 17dhC16 (Figure 4A),17dhC20 and 17dhC22, 10-fold increase in 17dhC22:1 (Figure 4B), and 5-fold increase in 17dhC24:1 (Figure 4C), among others. Likewise, treatment of Molt-4 cells with ST1926 resulted in 4-fold increase in 17dhC16 (Figure 4A), 2-fold increase in each of 17dhC20 and 17dhC22, 3-fold increase in 17dhC22:1 (Figure 4B), and 2-fold increase in 17dhC24:1 (Figure 4C), among others. The enhanced production of specific endogenous 17dhCer species indicated that ST1926 preferentially induces the activities of distinct CerS(s) in both tested cell lines, with the most likely candidates being CerS2, CerS4, CerS5, and CerS6 based on fatty acyl preferences of these enzymes [63]. In total, treatment with ST1926 elicited 3-fold and 4-fold increase in 17dhCer species in Molt-4 and HuT-102 cells over baseline, respectively (Supplementary Figure S1A). Meanwhile, there was no significant change in the ratio of most 17Cer/17dhCer species upon ST1926 treatment in both cell lines (Figure 5), indicating that ST1926 activates CerS(s), but does not inhibit the DEGS1, thus maintaining the increase of 17dhCer species that are being synthesized by CerSs and then DEGS1. Moreover, HuT-102 cells displayed lower basal levels of 17dhCer species compared to Molt-4 cells (Figure 4 and Supplementary Figure S1A), while no difference was observed in the basal level ratios of 17Cer/17dhCer species between the two cell lines (Supplementary Figure S1B). This is consistent with our previous results that HTLV-1 positive cells have a partial defect in Cer synthesis [13]. Indeed, these findings suggest that Tax oncoprotein has an inhibitory effect on CerS(s), and not the DEGS1.

### Treatment with ST1926 increases gene expression of ceramide synthases more prominently in HTLV-1 positive cells

**Figure 6:**
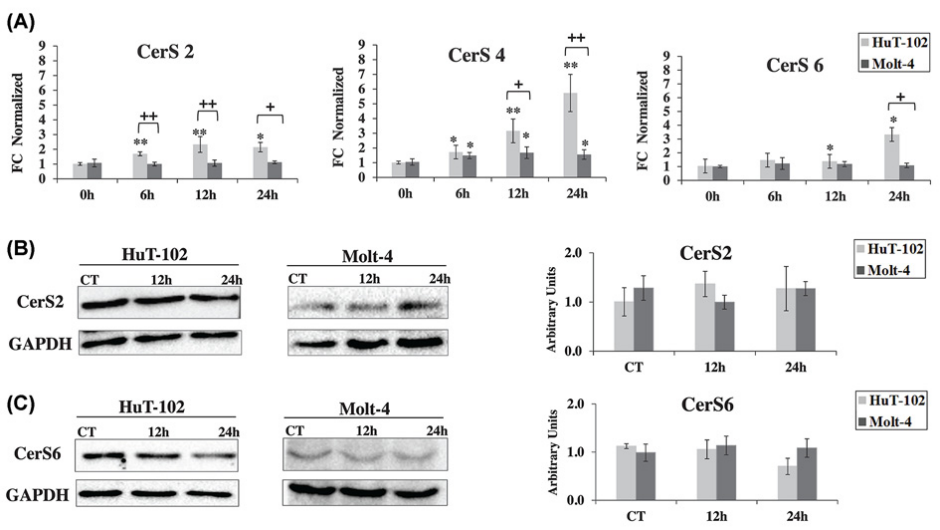
Effect of ST1926 on transcript and protein levels of ceramide synthases in HTLV-1 positive and negative malignant T cells (**A**) ST1926 increased gene expression levels of CerS2, CerS4, and CerS6 in HTLV-1 positive (HuT-102) and to a lesser extent in HTLV-1 negative (Molt-4 cells). HuT-102 and Molt-4 cells were seeded at a density of 3 × 10^5^ cells/ml and treated with 0.1% DMSO as control or 1 μM ST1926 for the times indicated. mRNA levels of CerS2, CerS4, and CerS6 were measured using qRT-PCR for the times indicated. The levels of mRNA were normalized relative to host β-actin. Fold change (FC) was quantified relative to cells treated with 0.1% DMSO as control. Data points represent the mean ± SEM. Results are representative of three independent experiments. The asterisks * and ** indicate statistically significant differences at *P*≤0.05 or *P*≤0.01, respectively, versus control using the *t*-test. Similarly, the plus sign ^+^ and ^++^ indicate statistically significant differences at *P*≤0.05 or *P*≤0.01 respectively, HuT-102 versus Molt-4 using the *t*-test. (**B**) ST1926 treatment had no effect on protein levels of CerS2 and CerS6 in HTLV-1 positive (HuT-102) and negative (Molt-4) cells. HuT-102 and Molt-4 cells were seeded at a density of 3 × 10^5^ cells/ml and treated with 1 μM ST1926 for the indicated timepoints. Whole SDS protein lysates (50 μg/lane) were prepared and immunoblotted against CerS2 and CerS6 antibodies. The blots were reprobed against GAPDH to ensure equal protein loading. Data points represent the mean ± SEM. Results are representative of three independent experiments.

While advances in spectrometric analyses facilitated the identification of Cer species, much less is known about the regulation of CerS(s). A better understanding of the regulation of CerS(s) is central to identify prognostic markers for different diseases and for the implementation of new treatments. In addition to enzyme activity, CerS(s) could be transcriptionally and/or translationally regulated [64]. HTLV-1 driven tumorigenesis is highly driven by Tax-mediated genomic instabilities that maintain pathogenesis and progression of the disease via pathways that regulate cell cycle, inhibit apoptosis, and favor survival [65,66]. Tax is required for malignant transformation of T cells and this is thought to be mediated through NF-κB activity [67]. We suspect that there is a cross-talk between NF-κB and Cer during ST1926-induced degradation of Tax and cell death. Tax, perhaps through NF-κB, may suppress the expression of some CerS(s) and once it is degraded the inhibition is released. Therefore, it was imperative to investigate the effect of ST1926 on the transcription of specific CerS(s) that are found to be highly expressed in leukocytes, namely CerS2, CerS4, and CerS6, in both HTLV-1 positive and negative malignant T cells [43]. We assessed CerS(s) status at mRNA and protein levels in response to treatment with 1 μM ST1926. Interestingly, we found a distinct time-dependent profile in mRNA levels of CerS2, CerS4, and CerS6 in HuT-102 treated cells, and to a lesser extent, in Molt-4 treated cells. In HuT-102 cells, ST1926 prompted a significant fold change in gene expression of CerS2 at 6 h (fold change = 1.7), CerS4 at 6 h (fold change = 1.7), and CerS6 at 12 h (fold change = 1.4), reaching to a fold change of 2, 5.7, and 3.3 at 24 h, respectively (Figure 6A). However, treatment of Molt-4 cells with ST1926 resulted in a less significant steady increase in gene expression of CerS4 than in HuT-102 (fold change = 1.7 at 12 h and fold change = 1.6 at 24 h), and no fold change in both CerS2 and CerS6 gene expression (Figure 6A). Meanwhile, treatment with ST1926 had no effect on the protein levels of CerS2 and CerS6 in both HuT-102 and Molt-4 cells (Figure 6B). Our reported data from LC/MS represent an indirect evidence that the activity of these CerS(s) is also increased by ST1926 in both HTLV-1 positive and negative malignant T cells. These results suggest that ST1926 plausibly regulates CerS(s) at the transcriptional and enzyme activity levels, without altering translational levels. Remarkably, the time-dependent increase in CerS(s) transcription levels in HuT-102, and not Molt-4, cells suggests that ST1926 relieves Tax-suppressive effects on Cer generation in HTLV-1 positive T cells.

### Bcl-2 delays cell death response and interferes with *de novo* ceramide synthesis induced by ST1926 in Molt-4 cells

**Figure 7:**
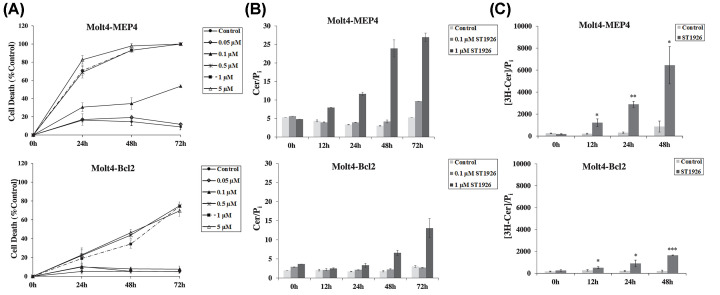
Bcl-2 interrupts ST1926-induced cell death and *de novo* ceramide accumulation in Molt-4 cells (**A**) Molt4-MEP4 and Molt4-Bcl2 were seeded at a density of 2 × 10^5^ cells/ml, treated with 0.1% DMSO or with increasing concentrations of ST1926 ranging from 5 × 10^−8^ to 5 × 10^−6^ M up to 3 days. Cells were assayed for death in quadruplicates using Trypan Blue Exclusion Assay. (**B**) Total ceramide (Cer) levels in Molt4-MEP4 and Molt4-Bcl2 at the indicated timepoints treated with 0.1 and 1 μM ST1926. Cer levels were determined using DGK assay as described in ‘Materials and Methods’ section and normalized to total cellular lipid phosphate levels. Data points represent the mean (± SD). Results are representative of two independent experiments. (**C**) Bcl-2 inhibits ST1926-induced *de novo* synthesis of Cer in Molt-4 cells. Cells were seeded at a density of 3 × 10^5^ cells/ml. *De novo* Cer levels were determined in triplicates using the [^3^H]-palmitate incorporation method as described in ‘Materials and Methods’ section and normalized to total cellular lipid phosphate levels. Data points represent the mean (± SD). The asterisks *, **, and *** indicate statistically significant differences at *P*≤0.05, *P*≤0.01, or *P*≤0.001, respectively, versus control using the *t*-test.

Based on cell type and stimuli, Cer coordinates a plethora of apoptotic pathways. Some of these pathways have been identified in hematological malignancies, whereby Cer mediates its apoptotic effects via its inhibitory action on Bcl-2 phosphorylation [34]. In other types of cancers, and based on the treatment used, Bcl-2 acts downstream of Cer, preventing Cer -mediated death in Molt-4 cells without interfering with its vincristine-induced accumulation [68]. Therefore, it became important to delineate the role of Bcl-2 in ST1926-initiated Cer production in cell death of malignant T cells. We treated Molt-4 cells overexpressing Bcl-2 (Molt4-Bcl2) and Molt-4 containing the empty vector p-MEP4 (Molt4-MEP4) with 0.1 or 1 μM ST1926 for 12, 24, 48, or 72 h. Cells were assayed for viability by Trypan Blue Exclusion Assay, while monitoring Cer accumulation upon treatment with the indicated concentrations and time-points. Bcl-2 attenuated the marked accumulation of Cer in Molt-4 starting at 24 h in response to ST1926 (Figure 7B). In particular, 0.1 μM ST1926 caused 50% cell death at 72 h, while no effect was observed in Bcl-2 overexpressing cells (Figure 7A). In addition, 1 μM ST1926 caused 70% and 20% cell death in Molt4-MEP4 and Molt4-Bcl2 cells, respectively (Figure 7A). Consistently, Bcl-2 interrupted early Cer accumulation at 12 h and blunted the level of accumulation up to 72 h (Figure 7B). One μM ST1926 caused a 2-fold increase in Cer as early as 12 h in Molt4-MEP4, while the same effect was delayed and observed in Molt4-Bcl-2 cells only after 48 h (Figure 7B). Moreover, treatment with 0.1 μM ST1926 showed no Cer accumulation up to 72 h in Molt4-Bcl2 cells, while causing a 2-fold increase in Molt4-MEP4 cells (Figure 7B). Our results indicate that Bcl-2 delays cell death-induced ST1926 treatment of malignant T cells and interferes with Cer generation. Since we have shown that ST1926 activates *de novo* pathway of Cer production (Figure 2), and that Bcl-2 interrupts Cer accumulation upon ST1926 treatment (Figure 7B), we wanted to investigate whether Bcl-2 has an inhibitory action on *de novo* generated Cer in response to ST1926 treatment in Molt-4 cells. We treated Molt4-MEP4 and Molt4-Bcl2 cells with 1 μM ST1926 for 12, 24, and 48 h in the presence of [**^3^**H] palmitate. Bcl-2 inhibited *de novo* Cer synthesis in response to ST1926 resulting in Cer levels that were several folds lower compared to control starting from 12 h post treatment (Figure 7C).

## Discussion

We report that ST1926 elicits early Cer accumulation in both HTLV-1 positive and negative malignant T cells. The kinetics and degree of Cer production show a unique response upon ST1926 treatment versus previously tested synthetic retinoids such as HPR, whereby Cer was only produced in HTLV-1 negative cells [13]. We have previously demonstrated that *de novo* Cer production is compromised in HTLV-1 positive cells probably due to the inhibitory action of Tax on CerS(s) activity in response to HPR [13]. Our results show that ST1926 concentrations as low as 0.1 μM increase Cer production and concomitantly reduce cell viability by 20% in HuT-102 cells [1]. ST1926 is a more potent inducer of cell cycle arrest and apoptosis than HPR or CD437, especially in HTLV-1 positive T cells, probably due to the fact that only ST1926 causes early reduction of Tax oncoprotein levels [1,8]. Among the previously mentioned synthetic retinoids, only ST1926 activates *de novo* Cer pathway in both HTLV-1 transformed and HTLV-1 negative malignant T cells. Interestingly, we have shown that early down-regulation of Tax oncoprotein by ST1926, but not HPR [13], precedes *de novo* Cer generation, which might be relieving the inhibitory effect of Tax on CerS(s). Therefore, it would be of interest to characterize the mechanisms by which Tax deregulates Cer pathways. Indeed, Tax disrupts major cellular processes, such as cell cycle, apoptosis, and cell proliferation [65]. Tax deregulates the NF-κB pathways [69,70], inducing the expression of genes that block apoptosis and might lead to chemoresistance, such as Bcl-2 [71–73]. Meanwhile, depending on stimuli and cell context, Bcl-2 is required for the inhibition of Cer synthesis [74] and resistance to permeabilization of mitochondrial membrane by Cer [75]. There is accumulating evidence that describes the relationship between Cer and NF-κB. Because Tax-mediated activation of NF-κB is central to transformation in HTLV-1-infected cells, we suspect that it may play a role in the inhibition of Cer. Alternatively, in HTLV-1 positive cells, Cer may exert some of its effects by inhibiting Tax-induced, NF-κB -mediated, anti-apoptotic pathways via activating various CAPPs such that, once Tax is degraded, the balance tips in favor of Cer and apoptosis proceeds. One probable explanation is that ST1926 degrades Tax and relieves its suppressive effect on Cer -generating pathway(s) in HTLV-1 positive malignant T cells. These inhibitory effects are in line with the established pro-apoptotic activities of Cer and the pro-survival, pro-proliferation, and pro-inflammatory effects of NF-κB. Interestingly, prediction studies using USCS Genome Browser suggest NF-κB transcription factor binding sites are present in the promotor regions of specific CerS(s) that generate Cer [64]. Therefore, the Tax-mediated activation of NF-κB and inhibition of Cer that promote transformation make it imperative to investigate their mutual relationships in this context and in response to ST1926 treatment as this may uncover novel approaches in the treatment of this highly malignant tumor.

It is well-established that HPR activates the rate-limiting enzyme, serine palmitoyltransferase [76–78]. Concurrently, HPR activates CerS, but inhibits DEGS1 in cell-free enzymatic assays as well as in intact cells, therefore, accumulating dhCer, but not Cer [77,78]. The effects of altered Cer levels have been widely studied in physiology and disease, with inadequate knowledge about the distinct Cer species. This might impede our understanding of the role of Cer, as most Cer species could be generated by at least two CerS(s). The epigenetic, transcriptional, translational, and post-translational regulation of the individual CerS(s) has been poorly investigated [64]. What is established is that CerS(s) regulate their activities by forming homo and heterodimers [79]. The activities of CerS(s) could also be regulated by phosphorylation, glycosylation, and acetylation to maintain protein stability [64,80,81]. Using LC/MS, we found that both HTLV-1 positive and negative T cell types markedly favor the accumulation of Cer versus dhCer molecular species upon treatment with ST1926. Specific Cer species, mainly MLC and VLC, preferentially accumulate in response to ST1926 treatment of HTLV-1 positive and negative T cells, indicating activity of the corresponding CerS(s), particularly CerS2, CerS4, CerS5, and CerS6. It has been shown that CerS5 mostly uses C16-CoA, and CerS6 mostly utilizes C14, C16, and C18-CoA, while CerS2 and CerS4 preferably use C20-C26 and C18-C20 fatty acyl CoAs, respectively [19,44,82,83]. The activation of these enzymes would explain our finding of increased 17dhC16, 17dhC20, 17dhC22, 17dhC22:1, and 17dhC24:1, among others, following treatment in the presence of 17C-sphinganine labeling. One possible explanation for specific species accumulation might be related to temporal regulation of CerS(s) activities, such that apoptotic cells mainly produce C16 and C18 Cers at the beginning of the apoptotic program [84,85], while C24-Cer predominantly accumulate during the final stages of apoptosis [85]. Interestingly, we found that HuT-102 and Molt-4 apoptotic cells mainly accumulate 17dhC16, that represents 48% and 71% of total 17dhCer species increase in HuT-102 and Molt-4 treated cells, respectively (Supplementary Table S1). Based on the accumulated Cer species in this work, our results suggest that specific CerS(s) might be involved in the Cer response to ST1926, plausibly through modulation of the CerS(s) enzymatic activity, without altering CerS(s) protein levels. Future experiments that perform *in vitro* CerS assays using different acyl-CoAs in cells treated with or without ST1926 would further validate our claim. We did find a differential CerS(s) gene expression response in the resistant and sensitive cells, whereby the expression of these specific CerS(s) genes was increased in Tax-positive cells (HuT-102) and, to a significantly lesser extent, in Tax-negative cells (Molt-4). This might be explained by the inhibitory effect of Tax on ceramide production. One of the deregulated mechanisms by Tax is the NF-κB pathway [65]. NF-κB might function upstream of ceramide, and this might be a plausible axis of regulation [86]. This highlights the importance of future mechanistic experiments that shall explain the interplay between Tax, NF-κB, and Cer, and verify the suggested inhibitory effect of Tax on CerS(s).

There is accumulating evidence that different fatty acyl chains generated by different CerS(s) play crucial roles in inducing cancer cell death and/or survival, depending on cell types and context [19,87]. Mecisek et al. reported that induction of *de novo* Cer synthesis is mediated by the activation of CerS 2, 5, and 6 isoforms in irradiated HeLa cells, and their subsequent interplay determines the balance between opposing anti- and pro-apoptotic Cer species [88]. The authors showed that while overexpression of CerS2 partially protected from irradiation (IR)-induced apoptosis, overexpression of CerS5 augmented the apoptotic effect in HeLa cells. The increase of C16-Cer in IR-induced Jurkat and HeLa cells has a pro-apoptotic role, while overexpression of CerS2, which generates C24-Cer, inhibits IR-induced apoptosis in these cells [45,88,89]. CerS expression is reduced by Fms-like tyrosine kinase 3 (FLT3) signaling in AML that confers resistance, while inhibition of FLT3 activity restores Cer synthase-induced mitophagy [90]. Interestingly, the pro-apoptotic effects of C16 and C18 Cers have been described in human leukemia cells and hematopoetic cells, respectively [45]. Moreover, CerS6 expression is increased in breast tumors, induces cell death in lung cancer cells, is elevated in breast tumors, and protects from graft-versus-host disease in a mouse model of leukemia [91–94]. However, it seems that the mere increase in a specific Cer species does not determine the cell fate [95]. Indeed, CerS co-transfection experiments in HCT116 cells and their effect on different Cer species production revealed that the equilibrium of medium, long, and very long ceramides committed cells to survival or death. Consistently, results have shown that VLCs, products of CerS2, inhibit mitochondrial C16-channel formation *ex vivo* and vice versa, which could also be regulated by Bcl-2 family proteins [96,97]. Whether alone or in combination treatment, such as with Bcl-2 selective inhibitors, ST1926 could be used to stimulate mitochondrial pathway of cell death. We have shown that Bcl-2 attenuates ST1926-induced cell growth arrest in Molt-4 cells. In addition, exogenous C6-Cer induced endogenous Cer-dependent cell death in these cells [68]. We observed early accumulation of Cer levels in Molt-4 cells by 12 h, which precedes cell death in these cells. However, Bcl-2 rescued Molt-4 cells from early Cer accumulation and cell death. Our results suggest a Cer-dependent mechanism of programmed cell death in response to ST1926 treatment, in which Bcl-2 acts upstream to prevent both Cer *de novo* synthesis and consequently, cell death in Molt-4 cells. This suggests that Bcl-2 and Cer act in a common pathway of the cell death mechanism, most probably with Cer acting in the ‘sensing’ phase rather in the ‘execution’ phase of apoptosis [68]. Therefore, these data might define a mitochondrial pathway of ST1926 action, with a critical inhibitory role of Bcl-2 in Cer production and cell death. Indeed, in some cancer systems, early Cer accumulation occurs downstream of Bcl-2 action and mitochondrial pathway, whereas in others Cer production occurs upstream of these actions. It is established that *de novo* Cer synthesis occurs in the endoplasmic reticulum as well as in the mitochondria and mitochondria associated membranes [98], wherein CerS(s) localize in both the outer mitochondrial outer and inner membranes. Therefore, drugs that would exclusively target a specific CerS and control apoptosis are potentially useful in chemoresistance. Given the diversity in regulating homo- or hetero-dimerization of different CerS(s) and the results presented in this research, it may be attractive to develop agents that allow the manipulation of essential apoptotic Cer acyl chain composition *in vivo* [79]. Further gene knockdown assays of the corresponding enzymes might help dissect these pathways in our and other systems. Interestingly, we observed that, compared with Molt-4, HuT-102 cells display lower basal levels of 17dhCer species, indicating a lower baseline activity of CerS, which elevate similarly in both cell lines upon ST1926 treatment. However, no difference is observed in the basal level ratios of 17Cer/17dhCer species between the two cell lines. This suggests that, under baseline conditions, Tax inhibitory effect is at the level of CerS and not the desaturase. Remarkably, these findings add to the novelty of the mechanism of action of ST1926 compared to the previously tested synthetic retinoids, HPR and CD437, whereby ST1926 relieves Tax inhibitory effect on CerS(s) and increases Cer levels.

One of the hurdles in cancer therapeutics is chemoresistance due to dysregulations in Cer metabolism [34]. In fact, decreased cellular Cer dictated by oncogene activation tilts sphingolipid metabolism to the production of prosurvival sphingolipids and correlates with tumorigenesis and drug resistance [17]. We have previously shown that the viral oncogene Tax could target the Cer pathway by inhibiting CerS activity, which might explain the resistance of HTLV-1 transformed cells to therapy [13]. We have also shown that Cer glucosylation by PDMP raises cellular Cer levels and increases the sensitivity of HTLV-1 positive T cells to HPR indicating that this approach may be therapeutically feasible [13]. Similarly to the inhibition of GCS, increasing endogenous Cer could be achieved by inhibiting its clearance by CDase, activation of sphingomyelin breakdown, and/or increasing *de novo* synthesis [17]. The detailed Cer analysis in the present study shows CerS as candidate enzyme that might be inhibited by Tax oncoprotein to particularly lower Cer levels in HTLV-1 positive cells, that is otherwise restored upon ST1926 treatment.

Therefore, novel approaches that target Cer metabolism might overcome drug resistance, plausibly by combining sphingolipid modulators with retinoids in order to provide novel approaches for treating cancer.

## Supplementary Material

Supplementary Figure S1 and Table S1

## Abbreviations

AML: acute myeloid leukemia
APL: acute promyelocytic leukemia
ATL: adult T-cell leukemia
ATRA: all-*trans* retinoic acid
CAPP: ceramide-activated protein phosphatases
CDase: ceramidase
CD437: 6-[3-(1-adamantyl)-4-hydroxyphenyl]-2-naphthalene carboxylic acid
CerS: ceramide synthase
DEGS1: dihydroceramide desaturase
DGK: diacylglycerol kinase
dhCer: dihydroceramide
DMSO: dimethylsulfoxide
FLT3: Fms-like tyrosine kinase 3
GCS: glucosylceramide synthase
HPR: N-(4-hydroxyphenyl) retinamide
HTLV-1: human T-cell lymphotropic virus-1
LC: long-chain
MLC: medium long-chain
NF-κB: nuclear factor kappa-light-chain-enhancer of activated B cells
PDMP: 1-phenyl-2-decanoylamino-3-morpholino-1-propanol
POLA1: DNA polymerase 1 alpha
PP1/PP2A: serine/threonine protein phosphatases
RRM: retinoid related molecule
SMS: sphingomyelin synthase
ST1926: (2E)-3-[3′-(1-adamantyl)-4′-hydroxy[1,1′-biphenyl]-4-yl]-2-propenoic acid
VLC: very long-chain
17Cer: 17C-ceramide
17dhCer: 17dhceramide
