# Supplementary material for: Restoration of ceramide de novo synthesis by the synthetic retinoid ST1926 as it induces adult T-cell leukemia cell death
Source: Biosci Rep. 2020 Oct 28;40(10):BSR20200050. doi: 10.1042/BSR20200050 (PMC7593536; doi:10.1042/BSR20200050)
Supplement: Supplementary Figure S1 and Table S1 [file BSR-2020-0050_supp.pdf]

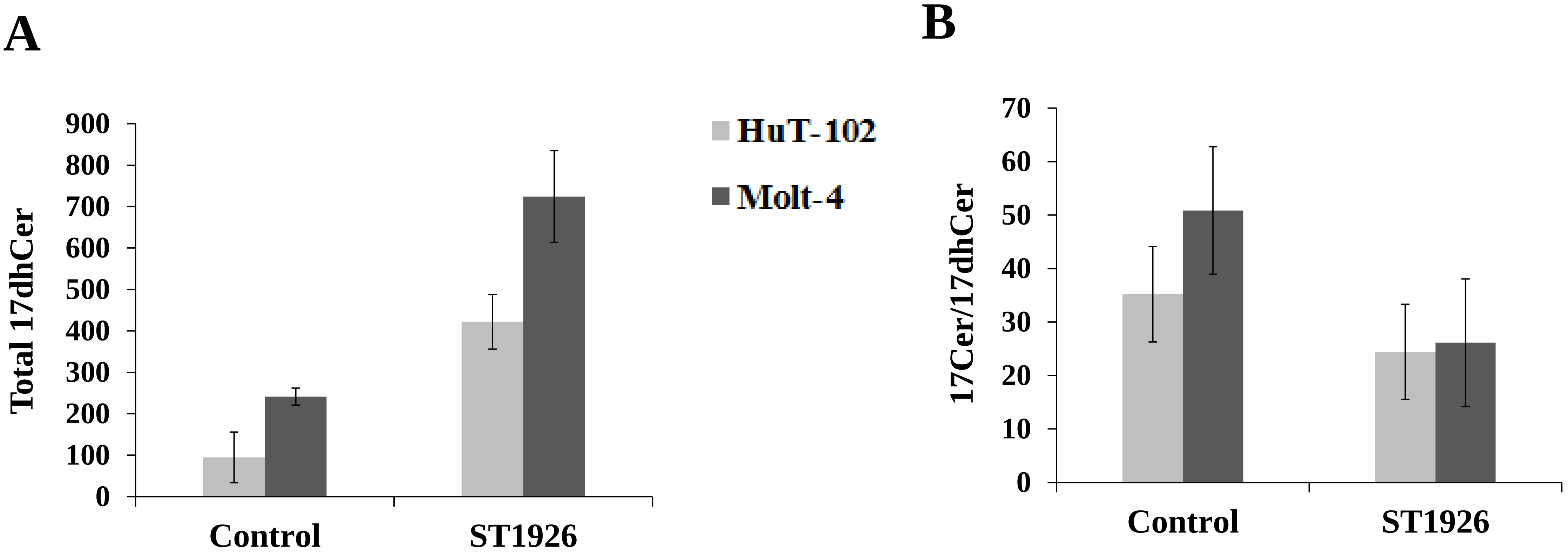

Supplementary Figure 1. Effect of ST1926 on total levels of 17dhCer, and ratio of total 17Cer to 17dhCer species in HTLV-1 positive and negative malignant T cells.

(A) HTLV-1 positive (HuT-102) cells has lower basal levels of 17C-dihydroceramide (17dhCer species) than Molt-4 cells, and treatment with ST1926 increased 17dhCer levels in both HuT-102 and Molt-4 cells, while **(B)** there was no significant change in the ratio of 17Cer/17dhCer species upon ST1926 treatment in both cell lines, or in the basal level ratios of 17Cer/17dhCer species between the two cell lines. Cells were seeded at a density of 3x105 cells/ml, labeled with 4 µM of unnatural 17C-sphinganine and treated with either 0.1% DMSO as control or 1µM ST1926 for 24h. 17dhCer species levels (pmol) were measured in lyophilized samples as duplicates by LC/MS as described in Methods and normalized to total cellular lipid phosphate levels (µmol). Data points represent the mean ± range (n=2). Results are representative of two independent experiments.

Supplementary Table 1

Percent increase in individual 17dhCer species in HuT-102 and Molt-4 cells

	Total 17dhCer	17dhC16 (% Total 17dhCer)	17dhC22 (% Total 17dhCer)	17dhC24:1 (% Total dhCer)
**HuT-102**	327	48	7	12
**Molt-4**	483	71	2	6

Supplementary Table 1. Percent accumulation of 17dhC16, 17dhC22, and 17dhC24:1 in HTLV-1 positive (HuT-102) and negative (Molt-4) malignant T cells, representing most prominent increase in their respective categories of medium long chain (MLC), long chain (LC), and very long chain (VLC) 17dhCer.
Cells were seeded at a density of $3x10^5$ cells/ml and treated with 0.1% DMSO as control or 1µM ST1926 for 24h. 17dhCer species levels (pmol) were measured in lyophilized samples as duplicates by LC/MS as described in Methods and normalized to total cellular lipid phosphate levels (µmol). Data points represent the mean $\pm$ range (n=2). Results are representative of two independent experiments.
